# Supplementary material for: Dopamine and glutamate regulate striatal acetylcholine in decision-making
Source: Nature. 2023 Aug 9;621(7979):577–85. doi: 10.1038/s41586-023-06492-9 (PMC10511323; doi:10.1038/s41586-023-06492-9)
Supplement: Supplementary file 1 — Supplementary Results, Discussion, Methods and References. [file 41586_2023_6492_MOESM1_ESM.pdf]

nature portfolio

https://doi.org/10.1038/s41586-023-06492-9

Supplementary information

Dopamine and glutamate regulate striatal acetylcholine in decision-making

In the format provided by the authors and unedited

Supplementary Results

Motivation for use of male mice

In our study, we exclusively used males because cholinergic signaling and its impact on behavior varies across males and females[1–6]. CINs may differentially regulate behavior across males versus females as exploratory behavior in males, but not females, is affected by loss of muscarinic Ach receptors on DANs in the ventral tegmental area[7]. Moreover, estrogen modulates cholinergic signaling through effects on choline acetyltransferase expression, Ach release, and choline transport[1–7].

Analysis of lateralization of DA and Ach signals

DA and Ach transients are similar for trials in which the mouse selected the port ipsilateral or contralateral to the fiber placement (**Extended Data Fig. 2e-h, 3d**). Differences between these trials were not statistically different for DA **(Extended Data Fig. 3e)**. Any significant differences for Ach were inconsistent in directionality, and the LDA classification accuracy was poor (**Extended Data Fig. 3f**); therefore, we combine data across both trial types.

ITI analysis of signals

To determine the extent to which the DA and Ach dynamics we observe are modulated by motor actions versus reward outcome and expectation, we analyzed center and side port entry signals that take place during the inter-trial-interval (ITI), during which reward is never delivered, and compared them to port entry signals that occur during unrewarded trials (**Extended Data Fig. 4b and 4c**). DA and Ach signals are modulated to a greater extent during unrewarded trials than during the ITI: DA signals dip more while Ach signals rise more after side port entry, and the opposite changes occur prior to side port entry. Likewise, DA and Ach dynamics diverge in complex ways upon center port entry during the ITI compared to during an unrewarded trial. Notably, center and side port entries themselves evoke very different DA and Ach dynamics during the ITI, indicating that the motor action of port entry alone is not sufficient to explain these signals. Altogether, this analysis reveals that, while the motor action may contribute to DA and Ach dynamics, reward expectation significantly shapes both neuromodulator transients.

GLM

The 2ABT is a complex behavior with multiple events that occur in quick succession and a GLM formally and quantitatively determines what behavior components can explain the observed signal. We define a set of behavior variables (such as timing of licks, port entries, and rewards) that the GLM can use to explain the neural data (**Extended Data Fig. 5a**). For each variable, the model assigns a kernel, comprised of a time series of β coefficients, that represent the time-dependent contribution of each behavioral variable to the photometry signal. Because of the small number of free parameters relative to the number of data points, the GLM can use an ordinary-least squares (OLS) cost function without regularization penalty terms (**Extended Data Fig. 5e**). To reconstruct the fluorescence transients, the kernels are convolved with the appropriate behavioral events and summed. If the GLM works well, the reconstructed signal recapitulates the measured signal well, which we can measure with the mean squared error (MSE, which is the cost function minimized by the model) between the two signals and the R^2 value of the fit (**Extended Data Fig. 5b**). Higher MSE and lower R^2, judged on test data not used for model training, indicate a greater difference between the real and predicted signal and thus poorer GLM performance. Note that the GLM can fit the trial-averaged signal perfectly without fully explaining trial-to-trial variability across this mean.

Cross correlation analysis of DA and Ach transients recorded from independent hemispheres

Comparison of the trial-averaged fluorescence signals suggest that increases in DA may suppress Ach with a short delay: an overlay of DA and Ach signals recorded from separate hemispheres reveals a striking reciprocal relationship between the two neuromodulators (**Extended Data Fig. 6a**). However, their overall reciprocity is accompanied by asynchronous fluctuations following side port entry, in which DA dips but Ach rises twice (**Extended Data Fig. 6a, orange arrows)**. This suggests a complex and potentially dynamic relationship between the two neuromodulators. Cross correlation analysis of these trial-segregated signals in which DA lags Ach reveals an anticorrelation with a positive time lag (**Extended Data Fig. 6b**), indicating that changes in DA are associated with delayed changes in Ach of the opposite sign. In addition, there is a smaller positive correlation at a negative time lag, suggesting that changes in Ach are followed by changes in DA of the same sign.

GLM with photometry variable

We quantified the interactions between simultaneously recorded DA and Ach signals using a GLM that incorporates photometry as a predictive variable alongside the behavioral variables, such that the dynamics in DA can be used to predict changes in Ach and vice versa. This model generated kernels of DA and Ach (**Fig. 2h**) which highly resemble the covariance function – both are dominated by a negative interaction occurring with time shifts of approximately 100 ms. Although the DA or Ach photometry signal alone is insufficient to robustly predict the other (**Extended Data Fig. 6h**) (DA GLM R^2 = 0.116; Ach GLM R^2 = 0.171), incorporation of these photometry kernels into history GLMs for DA and Ach improves model performance without overfitting (DA GLM R^2 = 0.336; Ach GLM R^2 = 0.388). This indicates that variation in one neuromodulator signal explains variation in the other signal that is not captured by the behavioral events, consistent with the existence of direct interactions between the two signaling systems.

Optogenetic activation of the CINs

To determine if CINs directly regulate striatal DA signals during decision making, we tested whether exogenous activation of CINs is sufficient to evoke DA release *in vivo* during decision making. First, we established conditions in which optogenetic activation of Chrimson-expressing CINs[8] triggers large and reliable Ach release (**Extended Data Fig. 7a**). Using these same stimulation parameters, we activated Chrimson-expressing or control mCherry-expressing CINs in the VLS of mice performing the 2ABT and simultaneously recorded DA release (**Extended Data Fig. 7b-d**). We demonstrated that CIN stimulation consistently and significantly evokes DA release (**Extended Data Fig. 7e**). Therefore, exogenous activation of CINs is sufficient to drive DA release. However, the increase in DA is small compared to the magnitudes of CIN-evoked DA observed *in vitro* and of reward-dependent DA release *in vivo*[9,10]. Consistent with this subtle effect on DA, we did not observe any effects on decision or side port occupancy time (**Extended Data Fig. 7f**).

GLM for DA and Ach signals upon D2R deletion in CINs

DA kernels are comparable across the three cohorts (**Extended Data Fig. 11c**). In contrast, all side-entry Ach kernels are greatly altered in Drd2-cKO mice, exhibiting an increase in β coefficients at negative time shifts across all histories as well as an increase in β coefficients at positive time shifts for histories with a rewarded outcome on the current trial

(**Extended Data Fig. 11b**) (WT DA GLM R^2 = 0.129; Drd2 f/f DA GLM R^2 = 0.122, Drd2-cKO DA GLM R^2 = 0.155; WT Ach GLM R^2 = 0.202; Drd2 f/f Ach GLM R^2 = 0.262, Drd2-cKO Ach GLM R^2 = 0.214). As a result, the relationship between Ach and DA signals in Drd2-cKO mice is severely disrupted (**Extended Data Fig. 11a**), with a significant reduction in the magnitude of their anticorrelation (**Fig. 4h; Extended Data Fig. 11e**) and the off-diagonal negative covariance (**Fig. 4i**).

Supplementary Discussion

CIN regulation of striatal DA

A mismatch in the firing of DANs and the release of DA in the striatum, notably during motivated approach behaviors and during reward value encoding by DA over extended timescales, suggests the potential for intra-striatal modulation of DA release[11]. Because synchronous CIN activation robustly triggers DA release *in vitro*, CINs are a prime candidate to drive DA release in striatum independently of the action potentials generated at DAN cell bodies. However, we did not find evidence that this effect is functionally important *in vivo* in the behavioral context of a reward-guided behavior: DA reward-encoding dynamics are unaffected by loss of Ach release in the VLS, and present even in the absence of Ach release throughout the entire striatum. Loss of CIN-mediated Ach release following chronic expression of tetanus toxin may lead to compensatory changes in the regulation of DA release. Although pharmacological inhibition of cholinergic effects on DAN axons using a β2 nAChR antagonist has been used to transiently inhibit this circuit, opposite conclusions of the effects on DA signals were observed across studies[12,13]. It will be informative to resolve these conflicting findings with alternate strategies that rapidly and reversibly inhibit CINs. Moreover, specific deletion of muscarinic and nicotinic Ach receptors in DANs are necessary to reveal if Ach can regulate other aspects of DA release. We found that optogenetic activation of CINs, despite being able to drive large transients in Ach levels, caused only small changes DA levels *in vivo* (e.g., compared to those evoked by rewards), which contrasts with the robust DA release evoked in striatal slices[9,10]. One caveat is that our method may not synchronously activate a sufficient population of CINs due to the spatial constraints of opsin expression and laser excitation. The discrepancy between the ability of CINs to regulate DA release *in vitro* and *in vivo* is surprising and may stem from fundamental differences between the two experimental systems. Within a striatal slice, basal neuromodulator levels are low, whereas *in vivo* CINs and DANs are both spontaneously active and constantly modulate their activity in response to environmental cues

and stimuli, which may create less permissive conditions for CINs to effectively drive DA release. Furthermore, *in vivo* CINs may be more inhibited via D2Rs, nAChRs may be more desensitized, or CIN activity may not be sufficiently synchronized across striatum. However, it remains possible that CINs can modulate DA levels to a greater extent in other *in vivo* contexts, for instance, set shifting[14] and reversal and extinction learning[15]. Finally, while we used male mice for our study, studies to determine if our results are generalizable to female mice will be informative.

DA regulation of striatal Ach

CIN pauses emerge after classical conditioning in response to salient and reward-predicting cues[16], and their coincidence with and dependence on DA release support the requirement for DA to generate this pause. However, a long-standing debate remains about whether DA is responsible for CIN pauses *in vivo*. Subsequent studies found that changes in DAN firing do not coincide with changes in CIN firing[17] and Ach pauses were partially suppressed, but not eliminated, upon D2R deletion in CINs[18]. Other inputs have been implicated in generating these pauses, including withdrawal of cortical inputs and excitation of thalamic neurons and GABAergic neurons from the VTA[19–21].

In the 2ABT, we observe Ach dynamics that are consistent with the classical CIN pause. Importantly, we find that Ach signals are reward responsive and sensitive to reward expectation, which contrasts with previous studies. This difference could stem from the fact that we measured neuromodulator levels instead of cell firing, and the former may be a more sensitive measure of changes induced by reward outcome. During a trial, we find that a subset of reductions in Ach transients cooccur with increases in DA levels and, contrary to prior findings[18], are eliminated when D2Rs are deleted in CINs. However, not all reductions in Ach signals are DA-dependent – only those that coincide with a rise in DA release. In fact, during unrewarded trials Ach levels dip alongside DA following side port entry and this transient is unaffected by D2R loss. Taken together, we posit that not all transient reductions in Ach levels are equivalent, and multiple mechanisms can generate them in a context-dependent manner. Some CIN pauses are DA-dependent, such as those in rewarded trials of the 2ABT, while others may partially depend on DA[18] or be DA-independent. In future studies, performing these D2R-deletions selectively in CINs in adulthood using a CRISPR-based approach will complement these results and address concerns of circuit rewiring or compensation.

Importantly, we also find that DA directly modulates Ach only at specific moments in time. First, DA inhibits Ach signals in a D2R-dependent manner at defined time points during a

trial - when DA is transiently elevated (**Fig. 4g**). This inhibition is not constitutive as implied in the circuit diagram. Without this D2R-dependent regulation, the anticorrelation that exists between DA and Ach signals is significantly reduced (**Fig. 4h**). Second, the anticorrelation between DA and Ach signals can be disrupted by mechanisms that remain to be determined. We performed a covariance analysis in which we calculate how the variance about the mean of DA at one time point influences the variance about the mean of the Ach signal at another time point (**Extended Data Fig. 6g**). This analysis reveals trial-by-trial variance in which DA and Ach signals are dominated by a strong negative correlation (**Fig. 2f**, see blue diagonal line). However, at a specific moment immediately after side port entry, this is transiently disrupted (**Fig. 2f,** see inset). We speculate that unknown inputs triggered by side port entry may override this underlying anticorrelation between DA and Ach. Altogether, these results demonstrate that transient DA release at specific moments within a trial inhibits Ach, which contributes to an anticorrelated relationship between DA and Ach. This anticorrelation can in turn be momentarily disrupted via other mechanisms that remain to be determined.

Extra-striatal regulation of Ach release

Another long-standing debate has been the role of cortex versus thalamus on CIN activity. Some studies support the thalamus as being the dominant input to CINs. Optogenetic studies revealed strong thalamic but negligible cortical connectivity onto CINs[22], and cortical activity did not correlate with striatal CIN activity in large scale recordings of mice performing a visually guided task[23]. In contrast, other studies have found that both the cortex and thalamus can drive CIN activity *in vitro*[24] and *in vivo*[25], and a rabies-based anatomical study revealed that CINs receive extensive inputs from both regions[26]. To address these divergent findings, we silenced each input individually using tetanus toxin. We found that loss of either cortical or thalamic transmission results in severe suppression of Ach levels and disruption of its release patterns; thus, both inputs are important modulators of CINs in the VLS. With our strategy, we cannot determine the relative contribution of each input to CIN activity because the degree of tetanus inhibition may vary due to differences in viral infectivity and toxin efficacy across cell types. Furthermore, disruption of each input affects many cells in the striatum (and in other brain regions) such that some of the effects on Ach levels may be indirect. While our study focused on the VLS, given the spatial and functional heterogeneity of striatum, a comprehensive survey of these inputs across different striatal regions is necessary to resolve different conclusions across past studies.

Contributions of Ach release to behavior

From our study, we gained insight into the multitude of complex interactions that take place in striatum. Nevertheless, much more remains to be understood about how neurotransmission is integrated across time and space to direct striatal function. Glutamate-driven phasic Ach elevations during side port entry and reward consumption suggest a role for this neuromodulator in regulating these key behavior events. Ach release has long been associated with movement, and Ach release in the 2ABT may directly drive key movements of mice into and out of the side port and their sustained licking bouts during reward consumption. Alternately, Ach release may reflect changes in the salience associated with these behavior events. Future experiments to precisely manipulate different periods of Ach release coupled with high-resolution behavior tracking will reveal how Ach regulates these behaviors. We are also interested to uncover mechanisms that modulate phasic glutamate release from the cortex and thalamus throughout the consumption period of rewarded trials, and what in turn causes glutamate release to transiently drop in unrewarded trials. Identification of the upstream input(s) that alter the activities of cortex and thalamus to drive this differential release in glutamate will be key to understand what underlies important components of the decision-making process, such as action evaluation, learning, evidence accumulation, and behavioral policies.

It is critical to define if and how the varying dynamics of DA, Ach, and glutamate during a trial modulates striatal plasticity. CIN pauses are hypothesized to be time windows that allow DA to potentiate corticostriatal and thalamostriatal synapses[27]. Consistent with this, a precise *in vivo* coincidence of CIN pauses, DAN activation, and striatal spiny projection neuron (SPN) depolarization via glutamate release is required for long-term potentiation of corticostriatal synapses[28]. Interestingly, both glutamate and Ach are repressed in mice that are proficient in the 2ABT, suggesting that SPN plasticity may not be modulated once an animal has learned the task. Instead, the main role of the striatum in a proficient mouse may be to execute the actions in a manner modelled by the RFLR or an alternate pre-learned algorithm. It will be necessary to establish the dynamics of glutamate, DA, and Ach release as mice learn the 2ABT, during which striatal plasticity is likely to be crucial. Unique patterns of neuromodulator release could tune the plasticity of striatal synapses in distinct ways to drive behavior. During rewarded trials, DA and Ach are tightly anticorrelated, which could permit potentiation at multiple time points if glutamate is elevated. In unrewarded trials, DA and Ach release are repressed, which could in turn promote depression. With D2R loss, the temporal gating of DA by Ach is disrupted, resulting in aberrant Ach signaling that may inhibit plasticity by reducing glutamatergic transmission[29] and ultimately impair the ability of mice to incorporate past knowledge into current actions.

Supplementary Methods

Viruses

The following viruses were used, with source and titer indicated in parentheses:
AAV2/9 hSyn-dlight1.1 (Boston's Children's hospital core (BCH); 6E12 GC/ml)
AAV2/9 hSyn-dlight1.1 D103A (BCH, 2.5E12 GC/ml)
AAV2/9 hSyn-GRAB Ach3.0 (WZ Biosciences, 6.5E12 GC/ml)
AAV2/9 hSyn-GRAB Ach3.0 mutant (Vigene, 1E12 GC/ml)
AAV 2/9 hSyn-GRAB-rDA1h (WZ Biosciences, 1.9E13 GC/ml)
AAV2/1 hSyn-DIO-ChrimsonR-tdTomato (UNC Vector Core, 2E12 GC/ml)
AAV2/8 hSyn-SIO-TelC-mCherry (Janelia Viral Core, 2.2E12 GC/ml)
AAV2/rg hSyn-SIO-TelC-mCherry (Janelia Viral Core, 5.5E12 GC/ml)
AAV2/8 hSyn-DIO-mCherry (Addgene, 2.5E13 GC/ml)
AAV2/rg hSyn-DIO-mCherry (Addgene, 9E12 GC/ml)
AAV2/1 hSyn-DIO-stGtACR2-FusionRed (Addgene, 4.2E13 GC/ml)
AAV2/1 hSyn-SF-iGluSnFR.A1848 (Addgene, 3E12 GC/ml)
AAV2/1 hSyn-DIO-NES-jRcamp1b-WPRE-SV40 (Addgene, 4.5E13 GC/ml)
AAV2/1 hsyn-jGCaMP8s-WPRE (Addgene, 2.8E13 GC/ml)
AAV2/1 hsyn-jGCaMP8m-WPRE (Addgene, 2.0E13 GC/ml)

Primary antibodies

The following antibodies were used with the source and dilution indicated in parentheses:
goat anti-Choline acetyltransferase (Millipore Sigma #AB144P; 1:200)
mouse anti-tyrosine hydroxylase (Immunostar #22941; 1:1000)
chicken anti-GFP (Abcam ab13970; 1:1500)
rabbit anti-GFP (Novus Biologicals #NB600-308; 1:1000)
rabbit anti-mCherry (Takara Bio #632496; 1:1000)
rabbit anti-GFAP (Abcam ab7260; 1:1500)

Data availability

All original photometry and behavior data can be found in the following publicly available Dropbox link:

https://www.dropbox.com/scl/fo/7imtk7hewz3t6o78djeer/h?rlkey=ir8j3ow1v29vsdy9oj5u9aiie&dl=0

The code for photometry analysis can be found at the following link: https://github.com/lchantran/AchDA_manuscript_photometry

The code for behavior analysis can be found at the following link: https://github.com/lchantran/AchDA_manuscript_behavior

Behavior performance analysis

Several metrics of behavior were used to characterize performance in this task and evaluate the ability of a predictive model to capture these behavioral patterns. The first is trial-by-trial dynamics around block transitions, where we calculate the probability that the mouse (i) chose the high probability reward port (p(high port)), and (ii) switched between ports as a function of each trial position within a block (p(switch)).

The behavior was also modeled with the purpose of systematically characterizing normal and perturbed patterns of behavior across treatment groups. The above behavioral features are accurately captured by a recursively formulated logistic regression model (RFLR)[30], which requires three interpretable parameters to recapitulate mouse behavior. Given successful predictive accuracy across experimental conditions, we can inspect how the model captures changes in mouse behavior that result from neural perturbations. The RFLR predicts future choice via a weighted combination of choice history bias (i.e., perseveration, α), and a latent representation of evidence that gets updated by new action-outcome information on every trial (β) and decays across trials (τ). Maximum likelihood parameter estimates were found using the stochastic gradient descent optimization algorithm. Fits for α, β, τ were presented for each of the experimental groups. Comparison of parameter fits provides a method of evaluating consistency in the structure of the behavioral strategy, as defined by a relative influence of choice perseveration, current evidence, and previous evidence (i.e., history).

To quantify differences across mice in p(switch) and the time course of p(high port) following the rewarded port transition, we used single value metrics of maximum p(switch) and the time constant of p(high port), $\tau_{highport}$, respectively. Higher maximum p(switch) and lower $\tau_{highport}$ indicate the performance of the mice on the task following the rewarded port transition. Lower $\tau_{highport}$ means that the mouse more rapidly identifies the new highly rewarded port, and a higher maximum p(switch) indicates greater flexibility of behavior induced by the environmental change.

A two-sided t-test was used to denote the significance of mean ITI, decision time, lose-switch, win-switch, and mean left rates. These metrics are means drawn from a distribution that approximates normal according to the central limit theorem. A Wilcoxon Rank Sum test was used to assess significance for the τ, maximum p(switch) and RFLR coefficient values. Here, these metrics are based on nonparametric data derived from exponential curve fits (in the case of $\tau_{highport}$), single values measured from a time course (maximum p(switch)) and fits of a linear regression model (RFLR coefficients); thus, a t-test is not applicable. Finally, a nonparametric test was used to determine statistical significance of conditional switching probability between the experimental and control datasets as no assumptions could be made about the true distribution of this data. Resampling from each dataset was done to calculate the difference (Δ) between each parameter for the experimental and control groups. This was repeated 1000 times to generate a distribution of bootstrapped Δs from which a 95% confidence interval could be calculated. If the confidence interval of the bootstrapped Δs did not overlap with zero, which is our null hypothesis, it was annotated as significant.

Analysis of photometry data

Signal demodulation

The frequency modulated signals were detrended using a rolling Z-score with a time window of 1 minute (12000 samples). As the ligand-dependent changes in fluorescence measured *in vivo* are small (few %) and the frequency modulation is large (~100%), the variance in the frequency modulated signal is largely ligand independent. In addition, the trial structure is rapid with inter-trial intervals of < 3 sec. Thus, Z-scoring on a large time window eliminates photobleaching without affecting signal. Detrended, frequency modulated signals were frequency demodulated by calculating a spectrogram with 1 Hz steps centered on the signal carrier frequency using the MATLAB 'spectrogram' function. The spectrogram was calculated in windows of 216 samples with 108 sample overlap, corresponding to a final sampling period of 54 ms. The demodulated signal was calculated as the power averaged across an 8 Hz frequency band centered on the carrier frequency. No additional low-pass filtering was used beyond that introduced by the spectrogram windowing. For quantification of fluorescence transients as Z-scores, the demodulated signal was passed through an additional rolling Z-score (1 min window). In select analyses, (cross-correlation in **Fig. 2d**), the same

approach was used but with 72 sample windows with 36 sample overlap in the spectrogram to yield an 18 msec final data sample period.

To synchronize photometry recordings with behavior data, center port entry timestamps from the Arduino were aligned with the digital data stream indicating times of center-port entries. Based on this alignment, all other port and lick timings were aligned and used to calculate the trial-type averaged data shown in all figures. The Z-scored fluorescence signals were averaged across trials, sessions, and mice with no additional data normalization. To identify signals that occur during the ITI, we labeled time periods that occur between the side port exit of one trial and the center port entry of the next trial as the ITI. We then searched for center and side port entry events that occurred within the ITI but which were separated from other port entry and exits by at least 15 time steps (0.81 sec). These ITI center in and side in events were then analyzed as above and compared to transients evoked around trial-associated (i.e., non-ITI) unrewarded port entries.

Cross-variance analyses

Cross-correlations were calculated using the 'xcorr' function in Matlab on normalized data and with the "normalized" option on. This function calculates the cross-correlation (range: -1 and 1, with the extremes indicating perfect anti-correlation and correlation, respectively) for two signals as a function of a time shift in one signal relative to the other (**Fig. 2d and 2e**). Cross-correlations were performed on demodulated fluorescence signals from an entire recording session (i.e., not segregated by trial structure), in which a positive lag indicates effects of changes in DA on Ach and a negative lag indicates effects of changes in Ach on DA (**Fig. 2d**). As a control for the cross correlation, the data was shuffled in two ways. First, the covariance was calculated by comparing the Ach signal in one session to the DA signal from the same animal from a different session (**Fig. 2d, green trace**). As an additional control to test for spurious correlations, the DA fluorescence transient in a session was circularly permuted by a random number of time steps (between 1 and 10000), and the cross-correlation between the original Ach transient and the shifted DA transient was calculated (**Fig. 2d, orange trace**). Both approaches preserve the statistics and auto-correlation structure of each signal but destroys their common relationship to behavioral events.

Cross-correlations were also performed on the signal and noise components of the trial-segregated responses (**Fig. 2e**). For each trial, 40 data points before and 100 data points after

the event of interest (e.g., center port entry time in all the cross-correlations shown) were concatenated for all trials of interest (e.g., rewarded trials) in one session. The cross-correlations of two such trial-concatenated signals were calculated using xcorr function as described above. For noise correlations (**Fig. 2e**), the trial-averaged signal was subtracted trial-by-trial and the residuals were concatenated and treated as above.

We also calculated two-dimensional cross-covariance (**Fig. 2f**) from the residual signals following subtraction of the trial-averaged signals as follows:

$$K(t_1, t_2) = \frac{1}{n}\sum_{i=1}^{n}\left(f_1^i - \langle f_1(t_1)\rangle\right)\left(f_2^i - \langle f_2(t_2)\rangle\right)$$

with i indicating the trial number (from 1 to n) and f_1 and f_2 corresponding to the two signals analyzed. This corresponds to, for each value of t_1 and t_2, calculating the mean value of the product of the residuals of each signal (relative to its trial-averaged mean) at the corresponding time points.

Quantitative analyses of signals

To quantify DA signals for each mouse, the mean of the Z-scored signal in the designated time range before (“pre”) or after (“post”) side entry (0.918 second window, encompassing 17 data points for most analyses; 0.756 second window, encompassing 14 data points for analyses of and comparisons to ITI port entry evoked signals) was calculated (**Extended Data Fig. 3a**). To quantify Ach signals, the difference in the maximum and minimum signal in the same time window was calculated, which is referred to as ΔAch (**Extended Data Fig. 3b**). Because of the multiphasic dynamics of Ach transients, ΔAch more accurately captures the differences in signals across conditions compared to the mean. In contrast, the mean is a more robust metric than ΔDA to quantify DA signals independent of which DA sensor was used due to differences in the kinetics of dLight1.1 versus rDAh. For both DA and Ach, comparisons were performed between pairs of signals from the denoted conditions, which is represented as a connected pair of dots (**Extended Data Fig. 3a and 3b**). Open circles indicate that the difference between each pair is statistically significant ($p<0.05$, two-sided t-test). Standard deviation is indicated by the error bars. These analyses were performed for each individual mouse and across the hemispheres of a mouse when applicable. Paired t-tests were used to calculate statistical significance, unless otherwise noted.

This analysis was complemented with linear discriminant analysis (LDA) to evaluate the degree to which the photometry signals are segregated. The LDA is a supervised dimensionality reduction technique which projects two classes of data onto a new axis that minimizes their variance and maximizes the distance between their means (**Extended Data Fig. 3c**). The data was split into a 70% training and 30% test ('holdout') set. A LDA classifier is generated from the training set, which is then used to classify the test set. The % classification accuracy of the trials in the test set is indicated in parentheses next to each mouse identifier. If there are robust differences between two groups, the LDA classifier will accurately categorize the test set.

References

1. Gibbs, R. B. Fluctuations in relative levels of choline acetyltransferase mRNA in different regions of the rat basal forebrain across the estrous cycle: effects of estrogen and progesterone. *J. Neurosci.* **16**, 1049 (1996).
2. Gibbs, R. B. Effects of estrogen on basal forebrain cholinergic neurons vary as a function of dose and duration of treatment. *Brain Res.* **757**, 10–16 (1997).
3. Luine, V. & Hearns, M. Spatial memory deficits in aged rats: contributions of the cholinergic system assessed by ChAT. *Brain Res.* **523**, 321–324 (1990).
4. Gabor, R., Nagle, R., Johnson, D. A. & Gibbs, R. B. Estrogen enhances potassium-stimulated acetylcholine release in the rat hippocampus. *Brain Res.* **962**, 244–247 (2003).
5. O'Malley, C. A., Dean Hautamaki, R., Kelley, M. & Meyer, E. M. Effects of ovariectomy and estradiol benzoate on high affinity choline uptake, ACh synthesis, and release from rat cerebral cortical synaptosomes. *Brain Res.* **403**, 389–392 (1987).
6. Singh, M., Meyer, E. M., Millard, W. J. & Simpkins, J. W. Ovarian steroid deprivation results in a reversible learning impairment and compromised cholinergic function in female Sprague-Dawley rats. *Brain Res.* **644**, 305–312 (1994).
7. Razidlo, J. A. *et al.* Chronic loss of muscarinic M5 receptor function manifests disparate impairments in exploratory behavior in male and female mice despite common dopamine regulation. *J. Neurosci.* JN-RM-1424-21 (2022). doi:10.1523/JNEUROSCI.1424-21.2022
8. Klapoetke, N. C. *et al.* Independent optical excitation of distinct neural populations. *Nat. Methods* **11**, 338–346 (2014).
9. Threlfell, S. *et al.* Striatal Dopamine Release Is Triggered by Synchronized Activity in Cholinergic Interneurons. *Neuron* **2**, 58–64 (2012).
10. Cachope, R. *et al.* Selective Activation of Cholinergic Interneurons Enhances Accumbal Phasic Dopamine Release : Setting the Tone for Reward Processing. *CELREP* **2**, 33–41

(2012).
11. Mohebi, A. *et al.* Dissociable dopamine dynamics for learning and motivation. *Nature* **570**, 65–70 (2019).
12. Krok, A. C., Mistry, P., Li, Y. & Tritsch, N. X. Intrinsic reward-like dopamine and acetylcholine dynamics in striatum. 1–21
13. Liu, C. *et al.* An action potential initiation mechanism in distal axons for the control of dopamine release. *Science (80-.).* **375**, 1378–1385 (2022).
14. Aoki, S., Liu, X. A. W., Zucca, A., Zucca, S. & Wickens, J. R. Role of Striatal Cholinergic Interneurons in Set-Shifting in the Rat. **35**, 9424–9431 (2015).
15. Okada, K. *et al.* Enhanced flexibility of place discrimination learning by targeting striatal cholinergic interneurons. (2014). doi:10.1038/ncomms4778
16. Aosaki, T., Graybiel, A. & Kimura, M. Effect of the Nigrostriatal Dopamine System on Acquired Neural Responses in the Striatum of Behaving Monkeys. *Science (80-.).* **265**, 412–415 (1994).
17. Morris, G., Arkadir, D., Nevet, A., Vaadia, E. & Bergman, H. Coincident but distinct messages of midbrain dopamine and striatal tonically active neurons. *Neuron* **43**, 133–143 (2004).
18. Martyniuk, K. M. *et al.* Dopamine D2Rs Coordinate Cue-Evoked Changes in Striatal Acetylcholine Levels. *Elife* (2022).
19. Ding, J. B., Guzman, J. N., Peterson, J. D., Goldberg, J. A. & Surmeier, D. J. Thalamic gating of corticostriatal signaling by cholinergic interneurons. *Neuron* **67**, 294–307 (2010).
20. Zhang, Y. *et al.* Pauses in Cholinergic Interneuron Activity Are Driven by Excitatory Input and Delayed Rectification , with Dopamine Modulation Report Pauses in Cholinergic Interneuron Activity Are Driven by Excitatory Input and Delayed Rectification , with Dopamine Modul. *Neuron* **98**, 918-925.e3 (2018).
21. Brown, M. T. C. *et al.* Ventral tegmental area GABA projections pause accumbal cholinergic interneurons to enhance associative learning. *Nature* **492**, 452–456 (2012).
22. Johansson, Y. & Silberberg, G. The Functional Organization of Cortical and Thalamic Inputs onto Five Types of Striatal Neurons Is Determined by Source and Target Cell Identities Article The Functional Organization of Cortical and Thalamic Inputs onto Five Types of Striatal Neurons Is D. *Cell Rep.* **30**, 1178-1194.e3 (2020).
23. Peters, A. J., Fabre, J. M. J. J., Steinmetz, N. A., Harris, K. D. & Carandini, M. Striatal activity topographically reflects cortical activity. *Nature* **591**, 420–425 (2021).
24. Kosillo, P., Zhang, Y.-F., Threlfell, S. & Cragg, S. J. Cortical Control of Striatal Dopamine

Transmission via Striatal Cholinergic Interneurons. *Cereb. Cortex* **26**, 4160–4169 (2016).

25. Doig, N. M., Magill, P. J., Apicella, P., Bolam, J. P. & Sharott, A. Cortical and thalamic excitation mediate the multiphasic responses of striatal cholinergic interneurons to motivationally salient stimuli. *J. Neurosci.* **34**, 3101–3117 (2014).
26. Guo, Q. *et al.* Whole-brain mapping of inputs to projection neurons and cholinergic interneurons in the dorsal striatum. *PLoS One* **10**, 1–15 (2015).
27. Cragg, S. J. Meaningful silences: How dopamine listens to the ACh pause. *Trends Neurosci.* **29**, 125–131 (2006).
28. Reynolds, J. N. J. *et al.* Coincidence of cholinergic pauses, dopaminergic activation and depolarisation of spiny projection neurons drives synaptic plasticity in the striatum. *Nat. Commun.* **13**, (2022).
29. Pakhotin, P. & Bracci, E. Cholinergic Interneurons Control the Excitatory Input to the Striatum. **27**, 391–400 (2007).
30. Beron, C. C., Neufeld, S. Q., Linderman, S. W. & Sabatini, B. L. Mice exhibit stochastic and efficient action switching during probabilistic decision making. *Proc. Natl. Acad. Sci. U. S. A.* **119**, 1–12 (2022).
