# Supplementary material for: Dopamine and glutamate regulate striatal acetylcholine in decision-making
Source: Nature. 2023 Aug 9;621(7979):577–85. doi: 10.1038/s41586-023-06492-9 (PMC10511323; doi:10.1038/s41586-023-06492-9)
Supplement: Supplementary file 2 — Reporting Summary [file 41586_2023_6492_MOESM2_ESM.pdf]

nature portfolio

Corresponding author(s): Bernardo Sabatini
Last updated by author(s): Jul 5, 2023

Reporting Summary

Nature Portfolio wishes to improve the reproducibility of the work that we publish. This form provides structure for consistency and transparency in reporting. For further information on Nature Portfolio policies, see our Editorial Policies and the Editorial Policy Checklist.

Statistics

For all statistical analyses, confirm that the following items are present in the figure legend, table legend, main text, or Methods section.

n/a	Confirmed	
☐	☒	The exact sample size (*n*) for each experimental group/condition, given as a discrete number and unit of measurement
☐	☒	A statement on whether measurements were taken from distinct samples or whether the same sample was measured repeatedly
☐	☒	The statistical test(s) used AND whether they are one- or two-sided<br>*Only common tests should be described solely by name; describe more complex techniques in the Methods section.*
☐	☒	A description of all covariates tested
☐	☒	A description of any assumptions or corrections, such as tests of normality and adjustment for multiple comparisons
☐	☒	A full description of the statistical parameters including central tendency (e.g. means) or other basic estimates (e.g. regression coefficient) AND variation (e.g. standard deviation) or associated estimates of uncertainty (e.g. confidence intervals)
☐	☒	For null hypothesis testing, the test statistic (e.g. *F*, *t*, *r*) with confidence intervals, effect sizes, degrees of freedom and *P* value noted<br>*Give P values as exact values whenever suitable.*
☐	☒	For Bayesian analysis, information on the choice of priors and Markov chain Monte Carlo settings
☐	☒	For hierarchical and complex designs, identification of the appropriate level for tests and full reporting of outcomes
☐	☒	Estimates of effect sizes (e.g. Cohen's *d*, Pearson's *r*), indicating how they were calculated

Our web collection on statistics for biologists contains articles on many of the points above.

Software and code

Policy information about availability of computer code

Data collection	OlyVIA software was used to acquire histology images. MATLAB_R2017a, LabJack, and Arduino software were used to acquire photometry and behavioral data
Data analysis	MATLAB_R2017b, MATLAB_R2021b, and Python3.0 were used to analyze all the data presented.

For manuscripts utilizing custom algorithms or software that are central to the research but not yet described in published literature, software must be made available to editors and reviewers. We strongly encourage code deposition in a community repository (e.g. GitHub). See the Nature Portfolio guidelines for submitting code & software for further information.

Data

Policy information about availability of data

All manuscripts must include a data availability statement. This statement should provide the following information, where applicable:

- Accession codes, unique identifiers, or web links for publicly available datasets
- A description of any restrictions on data availability
- For clinical datasets or third party data, please ensure that the statement adheres to our policy

The data and code that supports the findings of this study are available upon request from the corresponding author.

Research involving human participants, their data, or biological material

Policy information about studies with human participants or human data. See also policy information about sex, gender (identity/presentation), and sexual orientation and race, ethnicity and racism.

Reporting on sex and gender	No human subjects were used.
Reporting on race, ethnicity, or other socially relevant groupings	n/a
Population characteristics	n/a
Recruitment	n/a
Ethics oversight	n/a

Note that full information on the approval of the study protocol must also be provided in the manuscript.

Field-specific reporting

Please select the one below that is the best fit for your research. If you are not sure, read the appropriate sections before making your selection.

☒ Life sciences ☐ Behavioural & social sciences ☐ Ecological, evolutionary & environmental sciences

For a reference copy of the document with all sections, see nature.com/documents/nr-reporting-summary-flat.pdf

Life sciences study design

All studies must disclose on these points even when the disclosure is negative.

Sample size	For all experiments, a sample size of at least three was chosen. We determined this to be sufficient based on low variability observed between the photometry signals and behavioral performance we recorded.
Data exclusions	Data was not excluded from the analysis.
Replication	For all experiments, a replica number of at least three was chosen. We determined this to be sufficient based on low variability observed between the photometry signals and behavioral performance we recorded.
Randomization	There was no requirement for randomization.
Blinding	In comparisons between different treatment groups, the animals were each assigned a unique identification number which did not reveal their treatment.

Reporting for specific materials, systems and methods

We require information from authors about some types of materials, experimental systems and methods used in many studies. Here, indicate whether each material, system or method listed is relevant to your study. If you are not sure if a list item applies to your research, read the appropriate section before selecting a response.

Materials & experimental systems

n/a	Involved in the study	
☐	☒	Antibodies
☒	☐	Eukaryotic cell lines
☒	☐	Palaeontology and archaeology
☐	☒	Animals and other organisms
☒	☐	Clinical data
☒	☐	Dual use research of concern
☒	☐	Plants

Methods

n/a	Involved in the study	
☒	☐	ChIP-seq
☒	☐	Flow cytometry
☒	☐	MRI-based neuroimaging

Antibodies

Antibodies used	goat anti-Choline acetyltransferase (Millipore Sigma #AB144P; 1:200)<br>mouse anti-tyrosine hydroxylase (Immunostar #22941; 1:1000)

	chicken anti-GFP (Abcam ab13970; 1:1500)<br>rabbit anti-GFP (Novus Biologicals #NB600-308; 1:1000)<br>rabbit anti-mCherry (Takara Bio #632496; 1:1000)<br>rabbit anti-GFAP (Abcam ab7260; 1:1500)<br>Multiple lots of each antibody were used for this manuscript, all of which had equivalent performance.
Validation	All antibodies were validated by the respective manufacturers to work for immunohistochemistry, as stated on their online product pages. The rabbit anti-mCherry was only stated as applicable for use in western blots, but we performed our own validation to confirm that it specifically detects mCherry expressed in mouse brains (i.e. no signal was detected in the absence of fluorophore expression or in the presence of non-mCherry fluorophores, notably GFP).

Animals and other research organisms

Policy information about studies involving animals; ARRIVE guidelines recommended for reporting animal research, and Sex and Gender in Research

Laboratory animals	The following mice lines were used: C57BL6/J (Jackson labs #000664); ChAT-IRES-Cre (Jackson labs #006410); DAT-IRES-Cre (Jackson labs #006660), Drd2loxP (Jackson labs #020631); Vglut2-IRES-Cre (Jackson labs #028863); Vglut1-IRES-Cre (Jackson labs #023527). All mice were bred on a C57BL/6J genetic background and heterozygotes were used unless noted. For behavior experiments, males at 6-8 weeks of age were used.
Wild animals	No wild animals were used.
Reporting on sex	For behavior experiments, males at 6-8 weeks of age were used. Only males were used to avoid any behavioral variation due to the estrous cycle in female mice and because of recent findings that only male behavior is affected by loss of muscarinic Ach receptors (reference: Razidlo, J. A. et al. Chronic loss of muscarinic M5 receptor function manifests disparate impairments in exploratory behavior in male and female mice despite common dopamine regulation. J. Neurosci. JN-RM-1424-21 (2022). doi:10.1523/ JNEUROSCI.1424-21.2022)
Field-collected samples	n/a
Ethics oversight	All animal care and experimental manipulations were performed in accordance with protocols approved by the Harvard Standing Committee on Animal Care, following guidelines described in the US NIH Guide for the Care and Use of Laboratory Animals.

Note that full information on the approval of the study protocol must also be provided in the manuscript.
